# Comparative Survival Benefits of Surgery and Adjuvant Chemotherapy in Neuroendocrine Carcinoma of the Gallbladder: A Population-Based Study with Insight into Future Personalized Therapeutic Approach

**DOI:** 10.3390/jpm13061009

**Published:** 2023-06-18

**Authors:** Jaffar Khan, Asad Ullah, Abdul Qahar Khan Yasinzai, Abdul Waheed, Kalyani Ballur, Thomas E. Dickerson, Kaleem Ullah, Christopher D. Mejias, Omer Saeed

**Affiliations:** 1 Department of Pathology and Lab Medicine, Indiana University School of Medicine, Indianapolis, IN 46202, USA; 2 Department of Pathology and Laboratory Medicine, Vanderbilt University, Nashville, TN 37232, USA; 3 Bolan Medical College, Internal Medicine, Quetta 83700, Pakistan; 4 Department of Surgery, San Joaquin General Hospital, French Camp, CA 95231, USA; 5 Medical College of Georgia, Augusta University, Augusta, GA 30912, USA

**Keywords:** gallbladder, neuroendocrine carcinoma, SEER, radiation, surgery

## Abstract

Background: Neuroendocrine carcinomas of the gallbladder (NECs-GB) are rare tumors, accounting for <0.2% of all neuroendocrine carcinomas of the gastrointestinal tract. They originate from the neuroendocrine cells of the gallbladder epithelium with associated intestinal or gastric metaplasia. The current study is the largest study from the SEER database on NECs-GB that aims to elucidate the demographic, clinical, and pathologic factors influencing the prognosis and comparative survival analysis of different treatment modalities. Methods: The data from 176 patients with NECs-GB was abstracted from the Surveillance Epidemiology and End Result (SEER) database (2000–2018). Multivariate analysis, non-parametric survival analysis, and a chi-square test were used to analyze the data. Results: NECs-GB had a higher incidence amongst females (72.7%) and Caucasians (72.7%). Most patients had surgery only (N = 52, 29.5%), (N = 40) 22.7% had chemotherapy only, and (N = 23) 13.1% had chemotherapy with surgery. Only (N = 17) 9.7% had trimodaltiy (surgery, chemotherapy, and radiation therapy), and for (N = 41) 23.3% the status of chemotherapy was unknown, and these cases had neither radiation nor surgery. Conclusion: NECs-GB more frequently affects Caucasian females after the 6th decade of life. The combination of surgery, radiation, and adjuvant chemotherapy was associated with better long-term (5 years) outcomes, while surgery alone was associated with better short-term (<2 years) outcome survival.

## 1. Introduction

Neuroendocrine neoplasms of the gallbladder are a heterogenous group of tumors that account for 0.5% of all neuroendocrine tumors and <2% of gallbladder carcinomas [1,2]. They usually arise from the stem cells of the gallbladder, becoming neuroendocrine cells later on during development [2]. NECs-GB are even rarer and only represent 0.2% of all neuroendocrine carcinomas of the gastrointestinal tract [3].

Poorly differentiated neuroendocrine carcinomas of the gallbladder usually present with abdominal pain, jaundice, and palpable abdominal mass and can lead to paraneoplastic Cushing’s syndrome [4]. Additionally, the radiological features of the NECs-GB are similar to any other gallbladder malignancy, making it extremely challenging to diagnose prior to the surgical resection of the gallbladder [5]. The prognosis of NECs-GB is very poor, with a median survival rate of only 8.9 months, which is lower than gallbladder adenocarcinoma [6]. To better understand this rare entity, we aim to identify factors associated with survival and different treatment strategies. Understanding the pathogenesis and clinical presentation of GB-NEC is needed to improve treatment outcomes for this disease. In this study, we utilized the SEER database to analyze demographics, treatment, and outcomes and investigated potential avenues for therapeutic advancement in GB-NEC.

## 2. Materials and Methods

SEER stat software version 8.4.0 was used to extract data from the SEER database. Using the International Classification of Diseases version 3 (ICD-O-3). The data was collected from 18 registries of the SEER database. The registries include Alaska Native Tumor Registry, Arizona Indians Tumor Registry, Cherokee Nation Tumor Registry, Connecticut Tumor Registry, Detroit Tumor Registry, Georgia Center for Cancer Registry, Greater Bay Area Cancer Tumor Registry, Greater California Registry, Hawaii Tumor Registry, Iowa Tumor Registry, Kentucky Tumor Registry, Louisiana Tumor Registry, New Jersey Tumor Registry, Seattle-Puget Sound Tumor Registry, and Utah Tumor Registry from SEER software (https://seer.cancer.gov/seerstat/, accessed on 5 March 2023). For the demographic data analysis, we extracted data for age, race, and gender. The clinical data that we analyzed included tumor size, lymph node metastasis, and metastasis to the lung bone, brain, and liver at the time of the diagnosis. For the treatment modality, overall survival, survival with surgery, survival with radiation therapy, and survival with chemotherapy survival analysis was done for age, gender, and race. The cases included in our study were previously confirmed genetically or histologically. The “type of reporting resource” for our cases included hospital inpatient/outpatient facility clinic, laboratory only (hospital or private), physician’s office/private medical practitioner, and nursing/convalescent home/hospice. Those cases with no microscopic examination and radiography without microscopic confirmation and only clinical diagnosis were excluded from the study. For survival analysis, we only analyzed cases in which the patient’s age was known, which was confirmed microscopically, and those cases that presented with malignant behavior. The cases excluded from our survival analysis were cases diagnosed by death certificate only, unknown age, cases confirmed on an autopsy only, and those alive with no survival time. Endpoints examined included overall survival, mortality, and 1, 2, 3, 4, and 5 years of cancer-specific survival.

The data was exported to SAS v9.4 for thorough descriptive analysis and Kaplan–Meier survival analysis. Data that were either labeled unidentified or missing were removed from the univariate Kaplan–Meier survival analysis. Additionally, Cox regression analysis was employed to explore potential factors influencing survival.

## 3. Results

Data from a total of 176 NECs-GB patients were abstracted from 2000–2018.

### 3.1. Demographical Characteristics of Entire Cohort

The mean age was found to be 66.9 years with a standard deviation (SD) of ±13.0 years. The youngest case was 32 years of age. Most, 53 (30.1%), of the cases were between the ages of 70–79 years, and 6 (3.4%) of the cases were between 32–39 years of age; 13 (7.4%) were between 40–49 years of age, 27 (15.3%) were between the ages of 50–59 years of age, 47 (26.7%) were between 60–69 years of age, and 30 (17.0%) were ≥80 years of age. Of the 176 patients included in the study, most, 128 (72.7%), were female, while 48 (27.3%) were male. Regarding race, 128 (72.7%) of the cases were White, 23 (13.1%) were Black, 22 (12.5%) were Asian or Pacific Islander, and 2 (1.1%) were American Indian or Alaska Native. The tumor size was known in only 70 (39.8%) of the cases, while unknown in 106 (60.2%) of the cases. The tumor size at the time of resection when known was <2 cm in 16 (22.9%), 2–4 cm in 26 (37.1%), and >4 cm in 26 (37.1%) cases, while in 2 (2.9%) cases there was no residual tumor (Table 1).

#### 3.1.1. Distant Metastases at the Time of the Diagnosis of Gallbladder Neuroendocrine Carcinoma

The status of metastases was unknown in 73 (41.5%) of the cases, while it was known in 103 (58.5%) of the cases. Where known, 54 (52.4%) had no metastasis, while metastasis to brain, liver, and lungs at a time of diagnosis were 1 (0.97%), 39 (38.0%), and 1 (0.97%), respectively. Bone and live metastases combined was found in 1 (0.97%) of the known cases, brain and liver metastases combined was found in 1 (0.97%) of the known cases, and liver and lungs metastases combined was found in 1 (0.97%) of the known cases (Table 2).

#### 3.1.2. Treatment Characteristics

Of 176 cases, 40 (22.7%) had chemotherapy only, whilst just 1 (0.6%) had chemotherapy with radiation, and 23 (13.1%) had chemotherapy with surgery. Only 17 (9.7%) had trimodality therapy (chemotherapy + radiation + surgery). In 52 (29.5%) of the cases, the status of chemotherapy was unknown, but these cases had surgery without radiation. In 41 (23.3%) of the cases, the status of chemotherapy was unknown, and these cases had neither radiation nor surgery. In just 1 (0.6%) of the cases, chemotherapy was given without radiation while the status of surgery was unknown. In just 1 (0.6%) of the cases, the status of chemotherapy was unknown, but this case had both radiation and surgery combined (Table 3).

#### 3.1.3. Survival by Demographics

**Figure 1:**
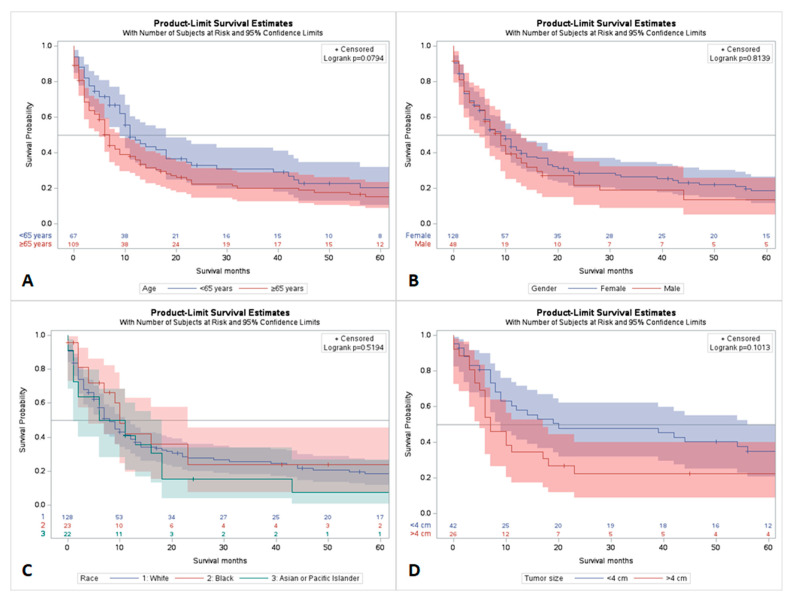
Comparative survival analysis of gallbladder NEC by demographics; (**A**) survival by age group; (**B**) survival by gender; (**C**) survival by race; (**D**) survival by tumor size.

Patients younger than 60 years of age had better short-term “<2 years” and long-term “>5” survival compared to those who were aged > 60 years. By comparing survival by gender, there was no survival benefit in the short-term, while there was a slightly higher long-term survival for females compared to their male counterparts. The Black race had a slightly short-term and long-term survival benefit as compared to White and Asian or pacific Islanders. The patients who had a tumor size greater than 4 cm had a decrease of both short- and long-term survival compared to those who had a tumor less than 4 cm in size (Figure 1).

#### 3.1.4. Survival by Different Treatment Modalities

**Figure 2:**
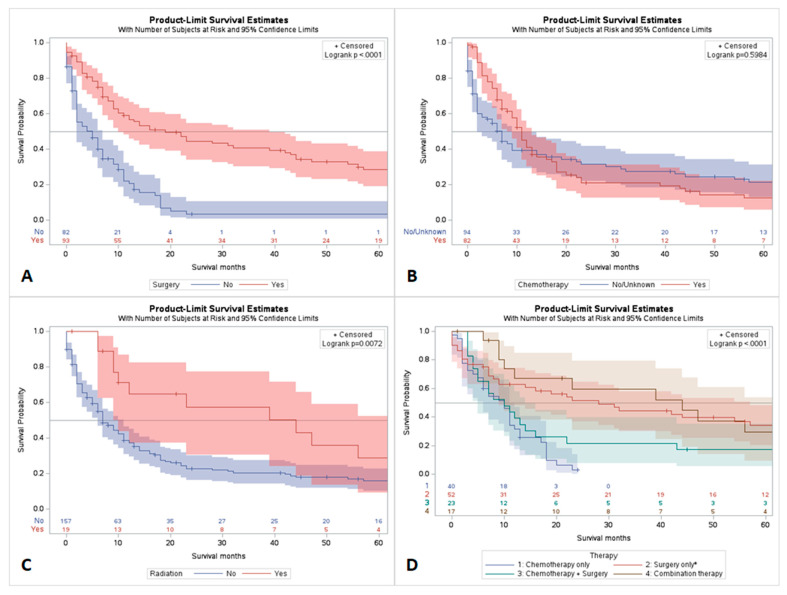
Comparative survival analysis of gallbladder NEC by different treatment modalities; (**A**) survival with surgery; (**B**) survival with chemotherapy; (**C**) survival with radiation therapy; (**D**) survival with combination (trimodality) therapies.

The best overall short-term survival was observed in patients who were treated with surgical therapy. Patients who were treated with chemotherapy had better short-term outcomes, while no survival benefit was observed for long-term chemotherapy. Those patients who were treated with combination therapy (surgery and chemotherapy) had better short-term and long-term survival outcomes as compared to single-modality treatment options (Figure 2).

## 4. Discussion

In this study, we found that gallbladder neuroendocrine carcinoma is more common in females. The combination therapy (surgery, radiation, and adjuvant chemotherapy) was associated with the overall best survival, while surgery alone was associated with the best short-term (<2 years) outcome.

Neuroendocrine neoplasms, first described by Oberndorfer et al. in 1907, are tumors that affect most epithelial organs in the body [2]. These neoplasms, although they share many similarities, have different clinicopathologic features as well as outcomes depending on the organ origin. In the past, different nomenclature and classification schemes existed for NENs depending on the organ system until the WHO published a unified classification system in 2018. Currently, neuroendocrine neoplasms are classified into two main categories: well-differentiated neuroendocrine tumors (NET) and neuroendocrine carcinomas (NEC). NETs are then graded into three grades depending on the mitotic count and/or Ki67 proliferation index. NECs are classified based on their morphology into small-cell and large-cell carcinomas.

Neuroendocrine neoplasms of the gallbladder, first described by Joel et al., are rare, accounting for 0.5–2% of all gallbladder cancers [7], and arise from Kulchitsky cells [2,8,9]. In the gallbladder, NECs are more common than NET [10]. While the etiology of NETs of the GB is unknown, that for NECs is postulated to be similar to that for GB adenocarcinoma such as gallstones [10]. Dasari et al. conducted a large data-based study about neuroendocrine tumors of the gallbladder, concluding that the incidence and prevalence of these tumors are on the rise, and survival for the neuroendocrine tumors has improved over time due to better treatment options [11]

The clinical manifestation of gallbladder NEC is not specific and can present with right upper quadrant pain, tenderness, and abdominal distention. However, these signs can be seen in cholelithiasis. Some cases of gall bladder NECs have been reported to present with carcinoid syndrome, but very rarely [12]. Imaging studies including CT, ultrasonography, and MRI can detect solid mass but are unable to differentiate between NECs and other types of gallbladder carcinomas. They can be helpful for staging as they can detect metastatic sites and lymph nodes. Currently, the diagnosis of NECs-Gb relies on the pathology result including the use of some immunohistochemical studies. The helpful biomarkers are Chromogranin A and synaptophysin, which are positive in 91.9% and 84.8%, respectively. In addition, the pathology result shows the grading and staging of these NECs-GB [13].

The pathogenesis of the NECs-GB is not well understood; however, various theories have been proposed. In a normal gallbladder, the presence of neuroendocrine cells is a rare phenomenon, but these cells are noticed in chronic cholecystitis gallbladder specimens [14]. The metaplastic changes in the gallbladder mucosa secondary to chronic cholecystitis might potentially explain the presence of these abnormal cells in the gallbladder mucosa [15]. Furthermore, some authors also believe that neuroendocrine cells in the gallbladder might be possible due to the undifferentiated gallbladder stem cells, which later on transform into neuroendocrine tumors [2]. Regardless of the various proposed mechanisms, the exact pathogenesis of NECs-GB remains unclear.

The standard therapeutic strategy with optimum survival benefits for NECs-GB is debatable; however, the surgical approach remains the gold standard management option [16,17]. There is no consensus on the treatment approach for gallbladder NEC up to this date. Ozer et al. studied a large cohort study of 6391 patients and suggested the consideration of adjuvant and neoadjuvant chemotherapy for gallbladder cancer. The study also suggested that adjuvant chemotherapy was associated with survival benefits in resectable gallbladder cancer, while neoadjuvant chemotherapy was associated with survival benefits in lymph node-positive gallbladder cancer [18]. However, to date, there are no treatment guidelines for NECs-GB due to the rare nature of the disease. Our study is the largest to highlight the comparative analysis between different treatment options and the short-term and long-term survival outcomes in NECs-GB. In our study, the surgical treatment improved short-term survival (<2 years), whereas the combination of surgery and adjuvant chemotherapy showed improved short- and long-term survival. As NECs-GB are rare tumors, therefore, no previous studies were available to compare the short-term and long-term survival for this entity. The use of neoadjuvant chemotherapy has several advantages, as suggested by Hakeem et al. [19]. The current study also revealed that the majority of the patients diagnosed with NECs-GB were treated with surgical resection (54%) with or without chemotherapy and radiation. Furthermore, 22.7% of patients received chemotherapy alone, with 13.1% treated with a combination of chemotherapy and surgery. The prognosis of gallbladder NEC is very poor. Duffy et al. reported that the median survival of gallbladder NEC was 9.8 months, which is lower than the median survival of gallbladder adenocarcinoma [6].

### Genomic Profiling and Future Personalized Therapy Approach

Variations in genomic profile aid in differentiating neuroendocrine carcinomas (NECs) and NETs and determining treatment response [20]. Common mutations reported in the literature involved in NECs include TP53, KRAS, and RB1, and these are associated with poor differentiation of the carcinoma [20]. TP53 and RB1 are mutated in about 80% of poorly differentiated neuroendocrine neoplasms (NENs), and data from mouse models indicate that the loss of these two genes is vital to the formation of poorly differentiated NENs [21]. These mutations can help differentiate NECs from well-differentiated high-grade NETs; this is especially applicable to pancreatic NENs. Additionally, these biomarkers can predict the response to platinum-based chemotherapy [20]. MEN1, DAX, and ATRX mutations are associated with well-differentiated neuroendocrine tumors (NETs) [22]. The presence of BRCA mutation in pancreatic NETs aids in determining the response to platinum-based therapy [18], whereas the presence of BRCA mutation in prostatic NECs aids in determining the response to poly [ADP-ribose] polymerase 1 inhibitors [20]. Lou et al. reported that in a group of 108 patients with confirmed pulmonary NECs, the anaplastic lymphoma kinase (ALK) fusion was reported in 0.9% [23]. The majority of neuroendocrine neoplasms (NENs) with ALK fusion are associated with metastasis, a higher grade, and a poorer prognosis, suggesting that pulmonary NECs with ALK fusion may indicate worse outcomes [20]. The neurotrophic receptor tyrosine kinase (NTRK) fusion is also associated with NETs [20]. According to Sigal et al., 6 out of 2417 patients with NET had NTRK fusions including intra- and inter-chromosomal translocations [2]. NTRK fusions in solid tumors are possible targets for therapies, such as entrectinib, but there is limited evidence for its use in NTRK fusion-associated NETs specifically [20]. A case report found that a patient with metastatic well-differentiated NET with the NTRK fusion was treated with entrectinib and demonstrated improved and protracted response [24].

Focusing on gallbladder NENs, the presence of protein kinase B, extracellular signal-regulated kinase, and the target protein of rapamycin are associated with poor prognosis [2]. VEGF plays an important role in the progression of gallbladder NENs [25] as well as in promoting angiogenesis, metastasis, and the recurrence of NENs [22]. Raymond et al. found that sunitinib, a tyrosine kinase inhibitor, improved progression-free survival in patients with pancreatic NENs by 11.4 months compared to 5.5 months in the placebo group [26]. The PI3K/AKT/mTOR pathway is involved in the progression of NENs [22]. Everolimus, a rapamycin inhibitor, has also shown improved progression-free survival and efficacy in patients with well-differentiated or moderately differentiated pancreatic NETs [27]. A study by Yao et al. found that the median progression-free survival in patients with low- or intermediate-grade pancreatic NETs with radiological progression in the past 12 months receiving everolimus was 11.0 months compared to 4.5 months in the placebo group [28]. Adverse effects of everolimus were mostly grade 1 or 2, with grade 3 or 4 events occurring at a rate of 6% in the everolimus group [28]. Everolimus can also be appropriate in patients with aggressive pulmonary NETs and less indolent GI NETS [29]. However, further research needs to be conducted to better understand the role of sunitinib as a potential treatment for patients with gallbladder NENs [2].

A study by de Bitter et al. analyzed biomarkers among patients diagnosed with high-grade neuroendocrine gallbladder cancer in the Netherlands between 2000 and 2019 [27]; 40% of their cases included mutations in RAD54L, ATM, or BRCA2, amplifications of ERBB2 or MDM2, or fusion of FGFR3-TACC3 [27]. These mutations are potential targets for personalized therapy and highlight the usefulness of molecular testing for patients with neuroendocrine gallbladder cancer. They found that the most commonly mutated genes were TP53 (70%), CTNNB1 (40%), RB1 (30%), and ATM (20%) [27]. Bitter et al.’s study included one case with the FGFR3-TACC3 fusion, which showed a response to FGFR therapy in a study by Voss et al. [30]. Their study consisted of 58 patients with FGFR-associated tumors treated with Debio 1347, an oral FGFR inhibitor, and they discovered that management with this inhibitor demonstrated acceptable tolerability, partial response in 6 patients, and size regression in 10 patients [30].

Accessed on 5 May 2023 https://www.ncbi.nlm.nih.gov/pmc/articles/PMC8529935/.

## 5. Limitations

Despite the finding mentioned above, our study has a few limitations, like most database studies. First, the information regarding the timing of the chemotherapy to the surgical resection (adjuvant vs. neoadjuvant) was not provided in the SEER database, limiting our interpretation of the results. Furthermore, there was also little information on the type of surgery and whether it was a simple cholecystectomy or a radical resection including a partial resection of the liver. Although we were able to assess tumor size, we were not able to assess to which layer of the gallbladder the tumor extended. The margin status is an important factor for survival, yet there was no data provided for margin status. Though in our study surgery is associated with the best short-term outcome, the margin status is important in the situation to further elaborate the prognosis. The dose of radiation was not provided in the SEER database, and there was no data available in SEER for radiation to see if the radiation was conducted for “intend to cure” or palliation. In addition, some of the critical clinical factors such as stage, socioeconomic factors, tumor grading, mitotic tumor index, and other associated pathologies that might affect the interpretations of the result were not coded correctly in the SEER database. Finally, the side effects of chemotherapy and radiation therapy were not available in the SEER database. Despite the limitations above, our study attempts to adequately describe the NENs-GB patient’s various less-understood clinical and demographical aspects.

## 6. Conclusions

Gallbladder neuroendocrine carcinomas are rare and aggressive tumors. In our study, a larger tumor size (>4 cm) and age >60 are associated with a worse prognosis. Patients with combination therapy such as surgery, radiation, and adjuvant chemotherapy had the best overall short- and long-term survival, while surgery provided the best short-term outcome. The current cohort is one of the most extensive databases studies related to this rare entity to the best of our knowledge. Even though the disease itself is rare, we could obtain a substantial number of cases. With the advent of novel chemotherapeutics, further analysis is required to account for the change in the prognosis of various subtypes. We suggest that an international registry enrolling all the NECs-GB should be introduced to understand this rare disease better.

## Figures and Tables

**Table 1:** Demographic profiles and tumor characteristics of patients with gallbladder neuroendocrine carcinoma.

Variable (*n* = 176)		Frequency (%)
age	32–39	6 (3.4%)
	40–49	13 (7.4%)
	50–59	27 (15.3%)
	60–69	47 (26.7%)
	70–79	53 (30.1%)
	≥80	30 (17.0%)
Gender	Female	128 (72.7%)
	Male	48 (27.3%)
Race	Unknown	1 (0.6%)
	White	128 (72.7%)
	Black	23 (13.1%)
	Asian or Pacific Islander	22 (12.5%)
	American Indian or Alaska Native	2 (1.1%)
Size	Unknown	106 (60.2%)
	Known	70 (39.8%)
	Size where known (*n* = 70)	
	No tumor found (0 cm)	2 (2.9%)
	≤2 cm	16 (22.9%)
	2–4 cm	26 (37.1%)
	>4 cm	26 (37.1%)

**Table 2:** Distant metastases at the time of diagnosis of patients with gallbladder neuroendocrine carcinoma.

SEER Metastasis (*n* = 176)	Frequency (%)
Unknown	73 (41.5%)
Known	103 (58.5%)
Where metastasis was known (*n* = 103)	
No metastases	54 (52.4%)
Brain metastasis only	1 (0.97%)
Liver metastasis only	39 (38.0%)
Lung metastasis only	1 (0.97%)
Bone + Liver metastases	1 (0.97%)
Brain + Liver metastases	1 (0.97%)
Liver + Lung metastases	5 (4.9%)
Bone + Liver + Lung metastases	1 (0.97%)

**Table 3:** Treatment characteristics of patients with gallbladder neuroendocrine carcinoma.

Treatment (*n* = 176)	Frequency (%)
Chemotherapy only	40 (22.7%)
Chemotherapy + Radiation	1 (0.6%)
Chemotherapy + Surgery	23 (13.1%)
Trimodaltiy therapy (Chemotherapy + Radiation + Surgery)	17 (9.7%)
Chemotherapy unknown|Radiation not done|Surgery done	52 (29.5%)
Chemotherapy unknown|Neither Radiation nor Surgery	41 (23.3%)
Chemotherapy done|Radiation not done|Surgery unknown	1 (0.6%)
Chemotherapy unknown|Both Radiation and Surgery done	1 (0.6%)

## Data Availability

All data are publicly available.
